# Same parasite, diverging fates: distinct responses of tenrecs to *Angiostrongylus cantonensis* infection

**DOI:** 10.1007/s11259-025-10870-1

**Published:** 2025-09-05

**Authors:** Anna Šipková, Petr Cibulka, Lucia Anettová, Divakaran Pandian, Jana Kačmaříková, Kristýna Javorská, Ladislav Novotný, David Modrý

**Affiliations:** 1 Department of Botany and Zoology, Faculty of Science, Masaryk University, Brno, Czech Republic; 2 Department of Veterinary Sciences, Faculty of Agrobiology, Food and Natural Resources/CINeZ, Czech University of Life Sciences, Prague, Czechia; 3 University of Veterinary Sciences Brno, Palackého tř. 1946/1, Brno, 612 42 Czechia; 4 Institute of Parasitology, Biology Center of Czech Academy of Sciences, České Budějovice, Czechia; 5 Department of Pathobiology, School of Veterinary Medicine, St. George’s University, True Blue, Grenada

**Keywords:** *Angiostrongylus cantonensis*, Invasion, Madagascar, *Echinops telfairi*

## Abstract

Tenrecs (Afrosoricida: Tenrecidae) are insectivorous mammals endemic to Madagascar, currently facing population declines due to habitat loss and subsistence hunting. Emerging infectious diseases, including parasitic infections, may pose additional threats. A comparable situation has been observed in Algerian hedgehogs (*Atelerix algirus*) in Mallorca, where the invasive nematode *Angiostrongylus cantonensis* has been associated with severe neuropathology. Given ecological parallels and the confirmed presence of *A. cantonensis* in Madagascar, this study aimed to assess its potential impact on tenrec health and survival. An experimental infection was conducted using *Echinops telfairi*, orally inoculated with 500 or 2000 third-stage larvae and monitored for 59 days through behavioral observations. Following euthanasia, artificial tissue digestion, qPCR analysis, and histopathology were performed. Baermann’s larvoscopy was used to examine feces from day 39 post-infection (DPI). No neuropathological symptoms were observed. Artificial digestion revealed 11 third-stage larvae in the gastrointestinal tract of one high-dose tenrec at 36 DPI. Parasite DNA was detected in various organs of both groups; however, accumulation in brain tissue occurred only in the high-dose group, with no viable larvae visible in histological sections. *E. telfairi* showed no apparent sensitivity to *A. cantonensis*, unlike the Algerian hedgehog, which develops severe neuropathology under comparable conditions. These findings suggest that *A. cantonensis* exhibits host-specific outcomes, and not all mammals act as aberrant hosts facing fatal infection. The persistence of third-stage larvae up to 36 DPI raises concerns about potential transmission to predators or hypothetical human infection.

## Introduction

Tenrecs (Afrosoricida: Tenrecidae) are insectivorous mammals endemic to Madagascar. This family exhibits remarkable morphological diversity, with species adapted to semifossorial, semiaquatic, terrestrial, and semiarboreal lifestyles. They primarily feed on insects and other invertebrates (Jenkins 2018). In Madagascar, tenrec populations are currently declining, primarily due to intensive deforestation and overhunting, as they serve as an important protein source, particularly in rural communities. Also, invasive pathogens, including parasites, may pose a further threat to tenrec populations (Annapragada et al. 2021; Stephenson et al. 2021).

The highest diversity of tenrec species is recorded in subtropical and tropical moist lowland and montane forests (as defined by IUCN 2025), which also provide suitable conditions for the circulation of invasive parasitic nematode *Angiostrongylus cantonensis* (Chen, 1935) (Metastrongyloidea: Angiostrongylidae) (Cowie et al. 2023). The parasite’s life cycle involves definitive hosts, primarily rats of the tribe Rattini, in which adult stages reproduce in the pulmonary arteries and heart. Various gastropod species serve as intermediate hosts (Cowie et al. 2023), where the larvae develop from the first to the third stage (Cowie 2013a). Due to the spread of invasive definitive and intermediate hosts, along with paratenic or transport hosts such as reptiles, amphibians, and crustaceans capable of harboring L3 (Anettová et al. 2022; Turck et al. 2022), A. *cantonensis* has successfully expanded its range across much of the tropical and subtropical regions worldwide, with high presence on humid tropical islands (Cowie et al. 2023). The presence of *A. cantonensis* in Madagascar has been documented in various regions, but only in definitive hosts, with no confirmed intermediate or paratenic hosts (Brygoo and Chabaud 1964; Alicata 1965, 1966; Breuil and Coulanges 1983), whereas *A. cantonensis* has been reported in rats and gastropods, as well as in human patients on nearby Indian Ocean islands such as Réunion, Mayotte, and Mauritius (Alicata 1966; Picot et al. 1976; Petithory et al. 1977; Graber et al. 1997; Epelboin et al. 2016).

Islands are renowned for their highly endemic and disharmonic biotas, in which invasive species often play unexpected ecological roles (Carlquist 1974; Drake et al. 2002; Whittaker and Fernández-Palacios 2007). Introduced rodents and gastropods can adopt novel ecological functions following colonization. This island phenomenon, associated with the introduction of *A. cantonensis* together with its hosts, was documented on Mallorca, where native Algerian hedgehogs *Atelerix algirus* (Lereboullet, 1842), were affected by the parasite and developed lethal neuropathological disorders (Paredes-Esquivel et al. 2019; Delgado-Serra et al. 2022).

The invasion of *A. cantonensis* into a new ecosystem requires the presence of suitable definitive and intermediate host species and their interaction in the foodweb. Successful invasion poses a potential risk to animal species that may accidentally ingest L3 in intermediate or paratenic hosts (Cowie 2013b) and subsequently act as aberrant hosts, in which *A. cantonensis* cannot complete its life cycle (Cowie 2013a). In such hosts, the larvae migrate to the central nervous system and induce severe eosinophilic meningitis, also known as neuroangiostrongyliasis (NA) (Cowie 2017; Jarvi and Prociv 2021; Cowie et al. 2022). The most well-documented aberrant hosts of *A. cantonensis* include humans, dogs, and non-human primates, as well as small mammals (such as hedgehogs and marsupials) and a range of avian species (Ma et al. 2013; Spratt 2015; Paredes-Esquivel et al. 2019; Cowie et al. 2022; Rizor et al. 2022).

Larger terrestrial species of tenrec (such as genera *Echinops*, *Setifer*, *Tenrec*, and *Hemicentetes*) exhibit ecological traits convergent to those of hedgehogs, suggesting a similar risk of infection with *A. cantonensis* (Jenkins 2018; Paredes-Esquivel et al. 2019; Delgado-Serra et al. 2022; Šipková et al. 2025). This study investigates the effects of *A. cantonensis* infection in tenrecs, using experimental infection of *Echinops telfairi* Martin, 1838 (the Lesser hedgehog tenrec) to determine whether this neurotropic nematode can induce pathology, potentially contributing to the gradual decline of tenrec populations on the island of Madagascar. Although no direct evidence currently links *A. cantonensis* to tenrec population declines, the overlap of ecological niches and abundance of reservoir hosts underscores the need for targeted surveillance and experimental investigation. Given the high diversity of tenrecs in habitats conducive to *A. cantonensis* transmission, and the absence of data on their susceptibility or potential role in the parasite’s life cycle, our study addresses an important knowledge gap.

## Materials and methods

### Experimental *A. cantonensis* strain and animal models

The *A. cantonensis* isolate used in this study originated from Tenerife, Canary Islands. The parasite was retrieved from *Insulivitrina lamarckii* (Férussac, 1821) collected in Tegueste (28.525340, -16.337740). The life cycle was maintained under laboratory conditions using laboratory rats (*Rattus norvegicus*, Wistar strain) and African giant snails (*Lissachatina fulica* (Férussac, 1821); Pulmonata: Achatinidae). The identity of the isolate was confirmed through morphological analysis of adult nematodes collected from infected rats and by cox1 gene sequencing, identifying the haplotype as belonging to *A. cantonensis* clade 2 (Červená et al. 2019), known also to infect hedgehogs in the Mediterranean (Paredes-Esquivel et al. 2019; Delgado-Serra et al. 2022). Experimental captive-born tenrecs *Echinops telfairi* (Afrosoricida: Tenrecidae) (7 males, 4 females, 12 months old) from a private breeder were acclimatized for 14 days before the start of the experiment. Parasitological screening using Sheather’s sugar flotation and Baermann’s larvoscopy showed no evidence of nematode infection.

The tenrecs were housed individually in plastic cages (floor area 4503 cm²) maintained at a constant temperature of 25 °C with access to natural daylight, monitored daily, and provided with *ad libitum* access to food; *Zophobas atratus* (Fabricius, 1775) larvae; and water. The cages were furnished with wood pellet bedding, shelters, branches, and wooden fibers. Two laboratory rats (*Rattus norvegicus*, Wistar strain) served as positive controls. These rats were obtained from the Laboratory Animal Breeding and Experimental Facility of Masaryk University and housed in plastic cages with a floor area of 2088 cm². Bedding consisted of wood pellets, and food and water were provided *ad libitum*. All experimental procedures followed stringent ethical and institutional guidelines and were approved by the Ministry of Education, Youth and Sports (No. MSMT-3967/2023-4).

## Experimental infection

**Fig. 1:**
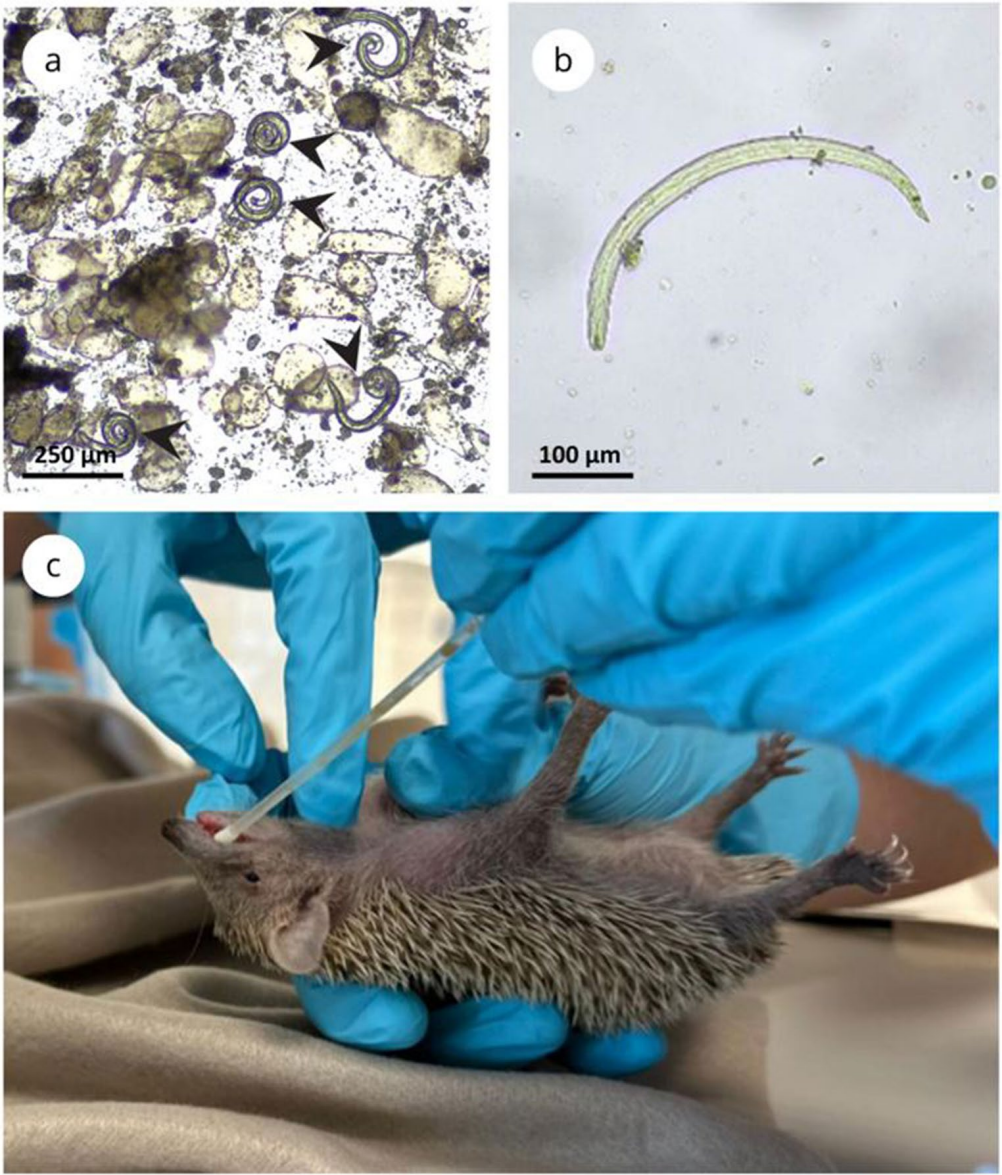
Experimental infection of *Echinops telfairi* by L3 of *Angiostrongylus cantonensis*. **a**: *A. cantonensis* L3 (marked with black arrows) recovered by artificial digestion of *Lissachatina fulica* muscle tissue for inoculum preparation; **b**: Dead L3 of *A. cantonensis* recovered from artificially digested gastrointestinal tissues of experimentally infected tenrec ETC1 (inoculated with 2000 L3); **c**: Experimental infection of *E. telfairi* with L3 of *A. cantonensis* via an oesophageal tube under isoflurane inhalation anaesthesia

Third-stage larvae of *A. cantonensis* were extracted from the tissue of experimentally infected *L. fulica* using artificial digestion. The snails were bred in the invertebrate facility at the University of Veterinary Sciences Brno. After decapitation, the foot muscle was processed in a digestive solution (0.3 g pepsin in 100 ml 0.7% HCl) on a magnetic stirrer at 600 rpm and 37 °C for two hours, as described in Modrý et al. (2021). The digested material was passed through a sieve into 50 ml Falcon tubes, followed by centrifugation (1 min, 1500 g). The resulting sediment was examined under a light microscope (40× magnification) in a Petri dish, and larvae were isolated using an automatic pipette and transferred into 2 ml tubes to prepare the inoculum (Fig. 1).

Three tenrecs in group A (individuals ETB1–ETB3) were each inoculated with 500 larvae via an oesophageal tube under isoflurane inhalation anaesthesia, while those in group B (ETC1–ETC4) received 2000 larvae per individual. The experiments were conducted in two phases, with group A being infected first, followed by group B. Due to the limited number of accessible animals, individuals were assigned to the experimental groups based on availability, without the application of predefined grouping criteria. Consistent experimental procedures and conditions were maintained across both groups. Four uninfected tenrecs (ETA1–ETA4) were included as negative controls, receiving saline solution via an oesophageal tube. These individuals were not sacrificed, as their primary purpose was to serve as behavioral controls for monitoring clinical or neurological changes in the infected groups. Due to ethical considerations, animals were returned to captive breeding afterwards. Two laboratory rats, used as positive controls, were inoculated in the same manner as the tenrecs but with a lower dose (40 L3) to avoid the development of clinical disease or mortality.

## Clinical examination

The animals were systematically monitored daily in the late afternoon to assess their food and water intake. Every other day, their mobility was assessed in a large plastic enclosure that allowed free movement to identify any behavioral changes. For five minutes, the animals’ ability to walk and respond to stimuli, such as mealworms, was evaluated; the eyes and nostrils were checked for any discharge. From 39 DPI onward, fecal samples were examined daily for the presence of L1 using Baermann’s larvoscopy. A comprehensive clinical examination and body weight measurement were performed under anesthesia at DPI 0 and at euthanasia (DPI 36–59). This evaluation included auscultation of the heart and lungs, palpation of the abdominal organs (liver and kidneys), and inspection of the oral cavity (teeth and mucous membrane color), anus, genital area, paws, and claws.

## Necropsy and histopathology

The tenrecs were euthanized using general isoflurane inhalation anesthesia, followed by intramuscular injection of dexmedetomidine (0.3 mg/kg, Orion Pharma Czechia) and ketamine (3 mg/kg, Bioveta a. s. Czechia). Euthanasia was completed with an intracardiac administration of T-61 euthanasia solution (0.3 ml/kg; embutramide, mebezonium iodide, tetracaine hydrochloride; MSD Animal Health). Upon euthanasia, the thoracic, abdominal, and oral cavities were opened and visually inspected for abnormalities. Tissue samples (Table 1) were placed in 2 ml tubes (stored at −20 °C) for qPCR analysis and in alcohol-formalin-acetic acid (AFA) fixative for histopathological examination, following the protocol described by Clopton (2004).

Histological preparations were conducted at St. Anne’s University Hospital in Brno. The fixed tissues were subjected to a series of dehydration steps using ethanol (70%, 90%, 96%), acetone, and xylene, and then embedded in paraffin. Tissue blocks were serially sectioned at 1.5 μm using a Leica RM 2010R microtome (Baria) and stained with H&E (Merck) using an E7 fully automated staining and coverslipping system (Bamed). For tissues where larvae were confirmed by qPCR (extrapolated larval count ≥ 1.00 per gram), additional histological sections were prepared. The slides were analyzed by a board-certified veterinary pathologist (LN) using a light microscope, equipped with a camera attachment. 3DHISTECH software was used to capture the images.

## Detection of nematode larvae in tissues by artificial digestion

The remaining tissues, excluding bones, were divided into three groups: (i) parenchymal tissues (lungs, liver, spleen, kidneys), (ii) gastrointestinal tract (stomach, small intestine, colon, rectum), and (iii) muscle tissues (gluteus, pectoralis, tongue, heart, diaphragm). Each group was subjected to artificial digestion in a digestive solution, as described above. The resulting sediment was transferred to Petri dishes and examined under a light microscope at 400× magnification for the presence of nematode larvae.

### DNA extraction, qPCR analysis, quantitative analysis

DNA was isolated from all sampled organs using the DNEasy Blood and Tissue Kit (Qiagen, Germany), which was optimized for L3 of *A. cantonensis* by prolonging the pre-lysis phase overnight. A quantitative PCR assay was conducted on a LightCycler 480 to detect *A. cantonensis* DNA in the tissues (Sears et al. 2021). The assay was performed in a 20 µl reaction mix containing 6.2 µl of 2x Mastermix (IDT Prime time gene expression master); 0.2 µl of 10 µM probe (PrimeTime Eco Probe 5′ 6-FAM/ZEN/3′ IBFQ,/56-FAM/ACA TGA AAC/ZEN/ACC TCA AAT GTG CTTCGA/3IABkFQ/); 0.8 µl of 10 µM primers (forward: AAA CTG TTG CTT TCG AAG, reverse: GCG CAA ATC TGA CGT TCT TG); 6.2 µl of DNA, DNase- and RNase-free water (PCR water) and 2 µl of DNA template. The cycling conditions (40 cycles) were set as follows: 95 °C for 20 s, 40 °C for 1 s and 60 °C for 20 s. DNA from a single *A. cantonensis* L3, processed in the same way, was used as a positive control, while PCR water served as a negative control. Samples were analyzed in duplicate, with Ct values ≤ 35 indicating a positive result. To quantify the number of larvae in the tissue samples, Ct values were converted to larval equivalents using a standard curve derived from serial dilutions (1×, 10×, 100×, and 1000×) of DNA extracted from a single L3. This standard curve was consistently used across all qPCR runs, with a 100× dilution of the positive control acting as the calibrator. DNA concentrations for each sample were then adjusted according to the weight of the extracted tissue and expressed as larval equivalents per gram (larvae eq./g tissue).

### Statistical analysis

Statistical analyses were performed using datasets containing tenrec body weights and *A. cantonensis* larvae equivalents per gram of tissue. A one-sided paired t-test was used to analyze significant weight differences within each experimental group separately, comparing weights before infection and on the day of euthanasia. A one-sided two-sample t-test was used to determine whether the weight differences between experimental groups were statistically significant (for this analysis, weight gain was calculated for each tenrec as final weight on the day of euthanasia minus initial weight before infection). To test for a monotonic increase in larvae equivalents per gram of brain tissue across groups (from the low-dose group A to high-dose group B animals euthanized at 36 DPI, and then to high-dose animals euthanized at 50 DPI), a Jonckheere–Terpstra trend test was performed. Statistical significance was defined as *p* < 0.05. All analyses were performed using R 4.3.1 (2023-06-16, “Beagle Scouts”, R Foundation for Statistical Computing) and RStudio version 2023.06.1 + 524.

## Results

### Presence of larvae in the gastrointestinal tract

Artificial digestion of gastrointestinal tissue from an ETC1 individual, euthanized at 36 days post-infection (DPI), revealed the presence of presumably dead larvae (Table 2; Fig. 1). The general morphology and length of the larvae (∼469 μm) were consistent with *A. cantonensis* L3 (Ash 1970). The presence of larvae in the gastrointestinal tract was further confirmed by detecting *A. cantonensis* DNA in stomach tissue (Table 1). No larvae were observed in other organs of ETC1 or any of the other tenrecs.

### Experimental tenrecs exhibited no signs of neurological disorder

None of the inoculated tenrecs exhibited neurological disorders directly attributable to *A. cantonensis* infection. Only exophthalmos was observed in individuals ETB1, ETC1, and ETC2; mild blepharoedema was observed in individual ETB3. In the absence of severe clinical manifestations, individuals in group A were euthanized at 59 DPI, whereas in group B, two individuals were euthanized at DPI 36 and the remaining two at DPI 50. Group A showed no statistically significant weight gain throughout the experiment (*p* ≈ 0.33), whereas Group B exhibited a considerable increase in weight post-infection (*p* ≈ 0.02). Although group B gained more weight on average than group A, the between-group difference did not reach statistical significance (*p* ≈ 0.07) (Table 2). All uninfected animals remained clinically healthy and maintained stable body weight throughout the experiment.

### Only highly infected tenrecs revealed the presence of parasite DNA in the brain

DNA was found in four peripheral organs: the stomach (ETC1), kidney (ETB2), liver, and ileum (ETB3). Examination of the brain revealed parasite DNA only in three out of four individuals of group B (Table 1). The Jonckheere trend test (*p* ≈ 0.02) indicates an increase in parasite DNA concentration in brain tissues, progressing from low-infected tenrecs (group A) to highly infected tenrecs euthanized at DPI 36, and further to those euthanized at DPI 50.

**Table 1:**
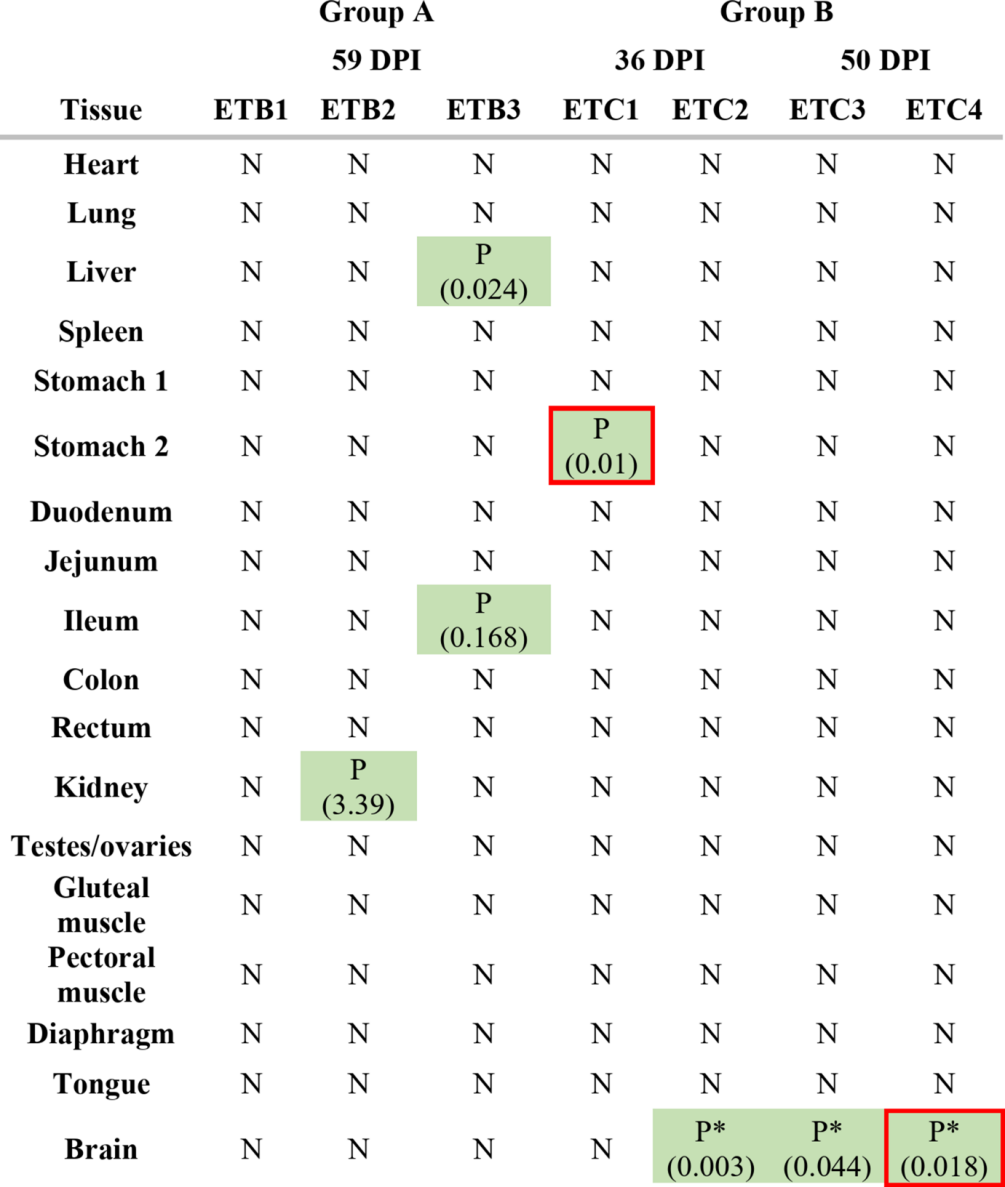
qPCR results from isolated tissues across Tenrec groups; green cells indicate *A. cantonensis* DNA-positive tissue samples, with numbers in brackets representing larval equivalents per gram of tissue (larvae eq./g tissue); red-bordered cells correspond to tissues where larvae or degraded larvae were found during artificial digestion or histopathological examination. An asterisk (*) denotes a statistically significant increase in parasite DNA concentration in brain tissues from low-infected Tenrecs (group A) to highly infected Tenrecs (group B), *p* ≈ 0.02

### Subtle brain granulomatous inflammation in highly infected tenrecs with multiple giant multinucleated cells

**Fig. 2:**
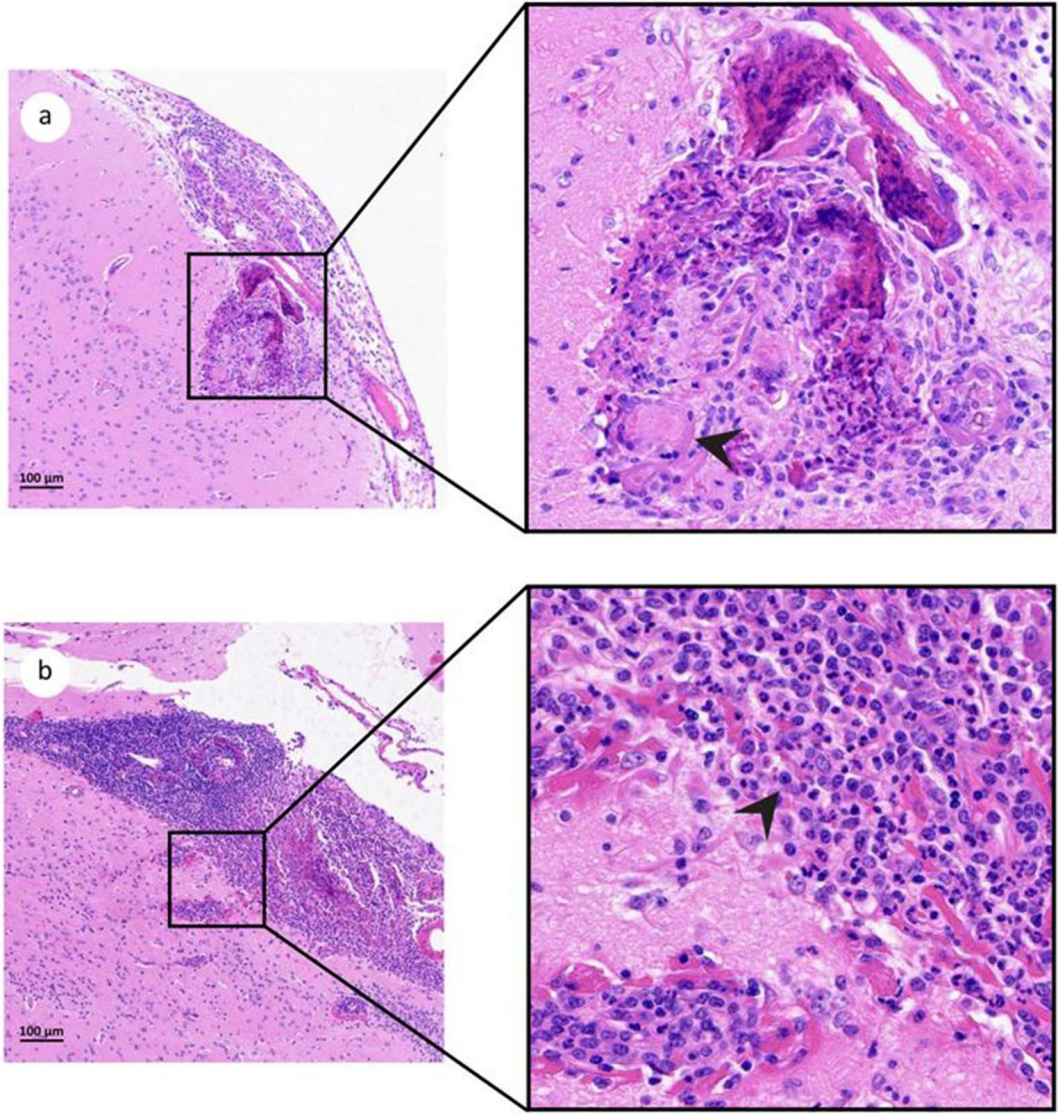
Brain sections of experimentally infected tenrecs (group B), inoculated with 2000 L3 of *A. cantonensis*, stained with H&E. **a**: Focal granulomatous meningitis with extension to the cerebral cortex of individual ETC4, detail showing multiple giant multinucleated cells marked with a black arrow; **b**: Lymphoplasmacytic and pyogranulomatous meningitis in individual ETC2, with a detail of the infiltrate composed of macrophages and neutrophils, indicated by a black arrow

Evidence of mild to moderate lymphoplasmacytic and pyogranulomatous meningitis was observed in highly infected individuals ETC1 and ETC2. In tenrec ETC4, sporadic granulomatous lesions were present in the cerebral cortex and hippocampus, accompanied by granulomatous meningitis with multiple giant multinucleated cells (Fig. 2; Table 2). The presence of giant multinucleated cells may indicate a reaction to degraded larvae. Additionally, moderate multifocal hepatocellular swelling was noted in the liver of the low-infected individual ETB1 (Table 2).

### Absence of L1 in tenrec feces

Coprological examination of fecal samples from all experimental tenrecs did not identify L1 of *A. cantonensis*. In contrast, numerous alive L1 were detected in the feces of the control Wistar rats, confirming the infectivity of the L3 in the original inoculum and their ability to complete the life cycle in a typical definitive host.

**Table 2:** Summary of clinical, histopathological examination, and artificial digestion, including weight differences and days of euthanasia. An asterisk indicates statistically significant weight increase, *: *p* ≈ 0.02. M, male; F, female

Tenrec	Group	Inoculum	Euthanasia [DPI]	Weight difference [g]	Clinical symptoms	Histopathology	Artificial digestion
ETB1 (M)	A	500 L3	59	−9	exophthalmos	moderate multifocal hepatocellular swelling	none
ETB2 (M)				10	none	none	none
ETB3 (M)				8	blepharoedema	none	none
ETC1 (M)	B	2000 L3	36	21*	exophthalmos	mild lymphoplasmacytic meningitis	11 dead L3
ETC2 (M)			36	5*	exophthalmos	moderate lymphoplasmacytic and pyogranulomatous meningitis	none
ETC3 (F)			50	12*	none	none	none
ETC4 (F)			50	27*	none	rare granulomas in the cerebral cortex and hippocampus and focally extensive granulomatous meningitis, with giant multinucleated cells	none

## Discussion

Emerging pathogens of wildlife stand among prominent examples of biological invasions, with numerous examples of their impact on biodiversity and contribution to species decline or even extinction (Thompson et al. 2014; Scheele et al. 2017; Dadam et al. 2019; Shanebeck and Lagrue 2020).

Compared to other pathogens, parasitic helminths are less frequently reported in the context of biological invasions, partly due to their complex life cycles. *Angiostrongylus cantonensis*, associated with highly invasive rats and gastropods, represents an exception, being reported as a detrimental wildlife pathogen in the invaded regions (Ma et al. 2013; Paredes-Esquivel et al. 2019).

Madagascar is an emblematic biodiversity hotspot with high levels of endemism; however, anthropogenic pressure, particularly habitat degradation and unsustainable hunting practices, has precipitated significant declines of native species (Ralimanana et al. 2022). Members of the Tenrecidae exhibit considerable morphological and ecological diversification, occupying a broad range of niches across the island (Jenkins 2018). Despite their adaptability, several tenrecid species are experiencing population decline, primarily because of habitat alteration (Annapragada et al. 2021; Stephenson et al. 2021). The introduction and spread of non-native invasive vertebrates further compound these threats, as they not only compete with native species for resources but also act as reservoirs and vectors of novel pathogens (Rasambainarivo and Goodman 2019).

Two species of invasive rats, *Rattus rattus* (Linnaeus, 1758) and *Rattus norvegicus* (Berkenhout, 1769), typical definitive hosts of *A. cantonensis*, have been introduced to Madagascar, and the populations are established across the island (Goodman 1995; Dammhahn et al. 2017; Rahelinirina et al. 2019). The transmission of pathogens by these invasive rodents in Madagascar has been well documented (Wilkinson et al. 2014; Dietrich et al. 2014). In parallel, the widespread establishment of invasive gastropods, notably *L. fulica*, a well-known intermediate host of *A. cantonensis* (Fontanilla et al. 2014; Lima et al. 2020), creates an environment for parasite circulation.

The most recent records of *A. cantonensis* on Madagascar date back to the 1980 s, when its presence in rats was described by Breuil and Coulanges et al. (1983). Occurrence of *A. cantonensis* in rats and gastropods has also been documented on nearby Indian Ocean islands, such as Réunion and Mauritius, along with cases of human infection observed in Mayotte and Réunion (Picot et al. 1976; Petithory et al. 1977; Graber et al. 1997; Epelboin et al. 2016; Balloy et al. 2024). Apparent circulation of *A. cantonensis* in rat-snail life cycle in Madagascar suggests the risk of exposure to *A. cantonensis* infection among native tenrecid species. A similar case was reported on the Balearic Islands, where lethal neuropathologies caused by *A. cantonensis* were documented in the population of Algerian hedgehogs (*Atelerix algirus*) in Mallorca (Paredes-Esquivel et al. 2019; Delgado-Serra et al. 2022; Arango-Colonna et al. 2023).

Our previous experimental study of *A. cantonensis* infection in hedgehogs (*Atelerix albiventris*) revealed a severe course of infection in individuals exposed to a high dose (2000 L3). Infected animals exhibited neurological symptoms such as circling and lateral instability, potentially fatal for individuals infected under natural conditions (Šipková et al. 2025). Based on findings in hedgehogs, this experimental study further investigates the consistency or variation of *A. cantonensis* infection and associated pathologies among small mammals ecologically similar to hedgehogs in regions endemic for *A. cantonensis*, using Lesser hedgehog tenrecs *Echinops telfairi* as a model.

Experimental inoculation of *E. telfairi* with *A. cantonensis* larvae demonstrates that even individuals exposed to high parasite loads (group B) exhibited no signs of neuropathology and maintained normal physiological condition throughout the study, with either stable or increased body weight. These results are contradictory to the clinical signs in hedgehogs infected with the same larval load (Šipková et al. 2025). Ingestion of *A. cantonensis* infective larvae by mammals is almost paradigmatically associated with the development of neurological disease. However, our experimental data from tenrecs suggest that this is not always the case. Apparently, it is not only the encounter filter but also differences in sensitivity among species that contribute to the spectrum of hosts affected by neuroangiostrongyliasis. The observed resistance of tenrecs to *A. cantonensis* infection is comparable to findings in other hosts, such as asymptomatic infections in experimentally inoculated pigs or total absence of clinically affected cats in hyperendemic regions where clinical cases are frequent among domestic dogs (Jindrák and Alicata 1968; Lee et al. 2021; Jarvi et al. 2024). In humans, high seroprevalence in endemic areas also suggests a high frequency of asymptomatic encounters (Chen et al. 2011; Cowie et al. 2022).

The absence of clinical signs in experimental tenrecs correlates with histopathological findings of mild to moderate meningitis and granulomatous reactions in highly infected individuals. These pathological findings may suggest reactions to larval degradation (Díaz et al. 2018). Liver alterations observed in one individual may be attributed to the migration route of the larvae and their subsequent death and degradation (Jindrák and Alicata 1968; Prociv and Turner 2018; Jarvi and Prociv 2021). Although the molecular analysis revealed the presence of *A. cantonensis* DNA in numerous tissues, the larvae were not observed, except for those isolated from gastrointestinal tissue. The presence of *A. cantonensis* DNA may be attributed to larval death and degradation, resulting in DNA release (Jarvi et al. 2015, 2024). Finding of dead L3 in the gastrointestinal tissues at DPI 36 suggests that larvae may survive within the gut wall for an extended period, which could have implications for the transmission of *A. cantonensis* to predators of tenrecs in Madagascar, such as domestic and feral dogs, the civets, fossas, and snakes (Jenkins 2018).

Several tenrecid species are also consumed by the local human population, particularly in rural areas, raising the potential risk of zoonotic transmission, especially if the animals are consumed undercooked or during meat preparation (Jenkins 2018; Annapragada et al. 2021; Cowie 2013b). This concern is further underscored by the frequency of neurological disorders reported among people in remote regions of the island (Gaud et al. 2014). To help clarify this potential risk and better understand parasite dynamics at the wildlife–human interface, we recommend the development of an integrated monitoring program involving parasitologists, conservation biologists, and public health authorities. While the present results offer valuable insights, they are based on a small cohort (*n* = 3–4 per group) with an unequal sex distribution. This limits statistical power and generalizability, and future studies should aim to include larger, sex-balanced groups to validate and expand upon these findings.

The newly obtained data indicate that *E. telfairi* does not act as a typical aberrant host, and infection with *A. cantonensis* does not appear to represent an additional threat to its populations. However, exposure to higher larval burdens from naturally infected gastropods (Tesana et al. 2009; Hollingsworth et al. 2013) could potentially trigger more severe pathological or clinical outcomes. Given the considerable species diversity within the family Tenrecidae, which comprises approximately 10 genera and over 30 species, it remains possible that other tenrec species in Madagascar may exhibit greater sensitivity to *A. cantonensis* infection, especially small-bodied members of the genus *Microgale*. Surprising resistance of Lesser hedgehog tenrecs to *A. cantonensis* infection demonstrates how limited our understanding of parasite-host interactions is in the case of this important zoonotic helminth.

## Data Availability

No datasets were generated or analysed during the current study.

## References

[CR1] Alicata JE (1965) Occurrence of *Angiostrongylus cantonensis* in madagascar, mauritius, ceylon, and Sarawak. J Parasitol 51:937 5848821

[CR2] Alicata JE (1966) The presence of *Angiostrongylus cantonensis* in Islands of the Indian ocean and probable role of the giant African snail, *Achatina fulica*, in dispersal of the parasite to the Pacific Islands. Can J Zool 44:1041–1049. 10.1139/z66-111 5981486 10.1139/z66-111

[CR3] Anettová L, Izquierdo-Rodriguez E, Foronda P, Baláž V, Novotný L, Modrý D (2022) Endemic Lizard *Gallotia Galloti* is a paratenic host of invasive *Angiostrongylus cantonensis* in tenerife, Spain. Parasitology 149:1–23. 10.1017/S0031182022000336 35321776 10.1017/S0031182022000336 PMC10090600

[CR4] Annapragada A, Brook CE, Luskin MS, Rahariniaina RP, Helin M, Razafinarivo O, Ambinintsoa Ralaiarison R, Randriamady HJ, Olson LE, Goodman SM, Golden CD (2021) Evaluation of Tenrec population viability and potential sustainable management under hunting pressure in Northeastern Madagascar. Anim Conserv 24:1059–1070. 10.1111/acv.12714

[CR5] Arango-Colonna M, Delgado-Serra S, Haines LR, Paredes-Esquivel C (2023) Improving the detection of *Angiostrongylus cantonensis* in the brain tissues of mammalian hosts. Acta Trop 242:106917. 10.1016/j.actatropica.2023.106917 37011831 10.1016/j.actatropica.2023.106917

[CR6] Ash LR (1970) Diagnostic morphology of the third-stage larvae of *Angiostrongylus cantonensis*, *Angiostrongylus vasorum*, *Aelurostrongylus abstrusus*, and *Anafilaroides rostratus* (Nematoda: Metastrongyloidea). J Parasitol 56:249–253 5445821

[CR7] Balloy L, Jacquart M, Binois F, Collet L, Traversier N, Daoudi J (2024) Cas d’angiostrongyloidose autochtone: Une émergence? Méd Mal Infect Form 3(Suppl):S109. 10.1016/j.mmifmc.2024.04.320

[CR8] Breuil J, Coulanges P (1983) Note Sur *Angiostrongylus cantonensis* a Madagascar. Arch Inst Pasteur Madag 50:35–38 7186336

[CR9] Brygoo ER, Chabaud AG (1964) Présence d’*Angiostrongylus cantonensis* à Madagascar. Ann Parasii Hum Comp 39:793

[CR10] Carlquist S (1974) Island biology. Columbia University Press, New York and London

[CR11] Červená B, Modrý D, Fecková B, Hrazdilová K, Foronda P, Alonso AM, Lee R, Walker J, Niebuhr CN, Malik R, Šlapeta J (2019) Low diversity of *Angiostrongylus cantonensis* complete mitochondrial DNA sequences from australia, hawaii, French Polynesia and the Canary Islands revealed using whole genome next-generation sequencing. Parasites Vectors 12:241. 10.1186/s13071-019-3491-y 31097040 10.1186/s13071-019-3491-y PMC6524341

[CR12] Chen MX, Zhang RL, Ai L, Chen JX, Chen SH, Huang DN, Gao ST, Geng YJ, Li XH, Zhu XQ (2011) Seroprevalence of *Angiostrongylus cantonensis* infection in humans in China. J Parasitol 97:144–145. 10.1371/journal.pone.0027747 21348622 10.1645/GE-2614.1

[CR13] Clopton RE (2004) AFA fixative protocol, from http://science.peru.edu/gregarina/Technique_Specimen.html#AFA_recipe. Accessed 16 July 2025

[CR14] Cowie RH (2013a) Biology, systematics, life cycle, and distribution of *Angiostrongylus cantonensis*, the cause of rat lungworm disease. Hawaii J Med Public Health 72(6 Suppl 2):6–9 23901372 PMC3689493

[CR15] Cowie RH (2013b) Pathways for transmission of angiostrongyliasis and the risk of disease associated with them. Hawaii J Med Public Health 72(6 Suppl 2):70–74 23901388 PMC3689478

[CR16] Cowie RH (2017) *Angiostrongylus cantonensis*: agent of a sometimes fatal globally emerging infectious disease (rat lungworm disease). ACS Chem Neurosci 8:2102–2104. 10.1021/acschemneuro.7b00335 28902487 10.1021/acschemneuro.7b00335

[CR17] Cowie RH, Ansdell V, Dunavan CP, Rollins RL (2022) Neuroangiostrongyliasis: global spread of an emerging tropical disease. Am J Trop Med Hyg 107:1166–1172. 10.4269/ajtmh.22-0360 36343594 10.4269/ajtmh.22-0360 PMC9768254

[CR18] Cowie RH, Malik R, Morgan ER (2023) Comparative biology of parasitic nematodes in the genus *Angiostrongylus* and related genera. Adv Parasitol 121:65–197. 10.1016/bs.apar.2023.05.003 37474239 10.1016/bs.apar.2023.05.003

[CR19] Dadam D, Robinson RA, Clements A, Peach WJ, Bennett M, Rowcliffe JM, Cunningham AA (2019) Avian malaria-mediated population decline of a widespread iconic bird species. R Soc Open Sci 6:182197. 10.1098/rsos.182197 31417708 10.1098/rsos.182197 PMC6689627

[CR20] Dammhahn M, Randriamoria TM, Goodman SM (2017) Broad and flexible stable isotope niches In Invasive non-native *Rattus* spp. In anthropogenic and natural habitats of central Eastern Madagascar. BMC Ecol 17:1–13. 10.1186/s12898-017-0125-0 28412938 10.1186/s12898-017-0125-0 PMC5393019

[CR21] Delgado-Serra S, Sola J, Negre N, Paredes-Esquivel C (2022) *Angiostrongylus cantonensis* nematode invasion pathway. Mallorca Spain Emerg Infect Dis 28:1163–1169. 10.3201/eid2806.212344 35608603 10.3201/eid2806.212344 PMC9155863

[CR22] Díaz Á, Sagasti C, Casaravilla C (2018) Granulomatous responses in larval taeniid infections. Parasite Immunol 40:e12523. 10.1111/pim.12523 29518254 10.1111/pim.12523

[CR23] Dietrich M, Wilkinson DA, Soarimalala V, Goodman SM, Dellagi K, Tortosa P (2014) Diversification of an emerging pathogen in a biodiversity hotspot: *Leptospira* in endemic small mammals of Madagascar. Mol Ecol 23:2783–2796. 10.1111/mec.12777 24784171 10.1111/mec.12777

[CR24] Drake DR, Mulder CPH, Towns DR, Daugherty CH (2002) The biology of insularity: an introduction. J Biogeogr 29:563–569. https://www.jstor.org/stable/827465

[CR25] Epelboin L, Blondé R, Chamouine A, Chrisment A, Diancourt L, Villemant N, Atale A, Cadix C, Caro V, Malvy D, Collet L (2016) *Angiostrongylus cantonensis* infection on Mayotte island, Indian ocean, 2007–2012. PLoS Negl Trop Dis 10:e0004635. 10.1371/journal.pntd.0004635 27144645 10.1371/journal.pntd.0004635 PMC4856411

[CR26] Fontanilla IKC, Sta Maria IMP, Garcia JRM, Ghate H, Naggs F, Wade CM (2014) Restricted genetic variation in populations of *Achatina* (*Lissachatina*) *fulica* outside of East Africa and the Indian ocean Islands points to the Indian ocean Islands as the earliest known common source. PLoS ONE 9:e105151. 10.1371/journal.pone.0105151 25203830 10.1371/journal.pone.0105151 PMC4159197

[CR27] Gaud S, Sauvée M, Debouverie M (2014) Pathologies neurologiques En milieu tropical et rural: expérience malgache d’un centre de Santé primaire de La région de Boeny. Med Sante Trop 24:312–316. https://www.jle.com/10.1684/mst.2014.0370 25296186 10.1684/mst.2014.0370

[CR28] Goodman SM (1995) *Rattus* on Madagascar and the dilemma of protecting the endemic rodent fauna. Conserv Biol 9:450–453

[CR29] Graber D, Jaffar-Bandjee MC, Attali T, Poisson J, Renouil M, Alessandri JL, Combes JC (1997) L’angiostrongylose Chez Le Nourrisson à La réunion et à mayotte. A Propos de Trois méningites à eosinophiles dont Une radiculomyéloencéphalite fatale avec hydrocéphalie. Arch Pediatr 4:424–429. 10.1016/S0929-693X(97)86666-3 9230991 10.1016/s0929-693x(97)86666-3

[CR30] Hollingsworth RG, Howe K, Jarvi SI (2013) Control measures for slug and snail hosts of *Angiostrongylus cantonensis*, with special reference to the semi-slug *Parmarion martensi*. Hawai’i J Med Public Health 72(6 Suppl 2):75–80 23901389 PMC3689477

[CR31] IUCN (2025) The IUNC N red list of threatened species. Version 2025-1. http://www.iucnredlist.org. Accessed 1 May 2025

[CR32] Jarvi S, Prociv P (2021) *Angiostrongylus cantonensis* and neuroangiostrongyliasis (rat lungworm disease): 2020. Parasitology 148:129–132. 10.1017/s003118202000236x 33315004 10.1017/S003118202000236X PMC11010204

[CR33] Jarvi SI, Pitt WC, Farias ME, Shiels L, Severino MG, Howe KM, Jacquier SH, Shiels AB, Amano KK, Luiz BC, Maher DE, Allison ML, Holtquist ZC, Scheibelhut NT (2015) Detection of *Angiostrongylus cantonensis* in the blood and peripheral tissues of wild Hawaiian rats (*Rattus rattus*) by a quantitative PCR (qPCR) assay. PLoS ONE 10:e0123064 25910229 10.1371/journal.pone.0123064 PMC4409314

[CR34] Jarvi S, Jacob J, Mina A, Lyons M (2024) Detection of rat lungworm (*Angiostrongylus cantonensis)* infection by real-time PCR from the peripheral blood of animals: a preliminary study. Parasitol Res 123:123–240. 10.1007/s00436-024-08251-9 38862687 10.1007/s00436-024-08251-9 PMC11166865

[CR35] Jenkins PD (2018) Family Tenericidae (tenrecs and shrew tenrecs). In: Wilson DE, Mittermeier RA (eds) Handbook of mammals of the world, 8. Insectivores, sloths and Colugos. Lynx Edicons, Barcelona, Spain, pp 134–173

[CR36] Jindrák K, Alicata JE (1968) Comparative pathology in experimental infection of pigs and calves with larvae of *Angiostrongylus cantonensis*. J Comp Pathol 78:371–382. 10.1016/0021-9975(68)90015-7 5691351 10.1016/0021-9975(68)90015-7

[CR37] Lee R, Pai TY, Churcher R, Davies S, Braddock J, Linton M, Yu J, Bell E, Wimpole J, Dengate A, Collins D, Brown N, Reppas G, Jaensch S, Wun MK, Martin P, Sears W, Šlapeta J, Malik R (2021) Further studies of neuroangiostrongyliasis (rat lungworm disease) in Australian dogs: 92 new cases (2010–2020) and results for a novel, highly sensitive qPCR assay. Parasitology 148:178–186. 10.1017/S0031182020001572 32829721 10.1017/S0031182020001572 PMC11010165

[CR38] Lima MG, Augusto RC, Pinheiro J, Thiengo SC (2020) Physiology and immunity of the invasive giant African snail, *Achatina* (*Lissachatina*) *fulica*, intermediate host of *Angiostrongylus cantonensis*. Dev Comp Immunol 105:103579. 10.1016/j.dci.2019.103579 31877327 10.1016/j.dci.2019.103579

[CR39] Ma G, Dennis M, Rose K, Spratt D, Spielman D (2013) Tawny frogmouths and brushtail possums as sentinels for *Angiostrongylus cantonensis*, the rat lungworm. Vet Parasitol 192:158–165. 10.1016/j.vetpar.2012.11.009 23218219 10.1016/j.vetpar.2012.11.009

[CR40] Modrý D, Fecková B, Putnová B, Manalo SM, Otranto D (2021) Alternative pathways in *Angiostrongylus cantonensis* (Metastrongyloidea: Angiostrongylidae) transmission. Parasitology 148:167–173. 10.1017/s0031182020001857 32981541 10.1017/S0031182020001857 PMC11010052

[CR41] Paredes-Esquivel C, Sola J, Delgado-Serra S, Riera MP, Negre N, Miranda MÁ, Jurado-Rivera JA (2019) *Angiostrongylus cantonensis* in North African hedgehogs as vertebrate hosts, mallorca, spain, October 2018. Eurosurveillance 24:1900489. 10.2807/1560-7917.es.2019.24.33.1900489 31431209 10.2807/1560-7917.ES.2019.24.33.1900489 PMC6702795

[CR42] Petithory J, Jay M, Colette S (1977) Premier Cas de méningite à éosinophiles á La réunion, probablement due a *Angiostrongylus*. Bull Soc Pathol Exot 70:151–155 579615

[CR43] Picot H, Lavarde V, Grillot ML (1976) Existence d’*Angiostrongylus cantonensis* a La réunion isolement de La Souche. Bull Soc Pathol Exot 69:329–331 1037434

[CR44] Prociv P, Turner M (2018) Neuroangiostrongyliasis: the subarachnoid phase and its implications for anthelminthic therapy. Am J Trop Med Hyg 98:353–359. 10.4269/ajtmh.17-0206 29210355 10.4269/ajtmh.17-0206 PMC5929180

[CR45] Rahelinirina S, Bourhy P, Andriamiaramanana F, Garin B, Rajerison M (2019) High prevalence of *Leptospira* spp. In rodents In an urban setting In Madagascar. Am J Trop Med Hyg 100:1079–1081. 10.4269/ajtmh.18-0642 30915950 10.4269/ajtmh.18-0642 PMC6493950

[CR46] Ralimanana H, Perrigo AL, Smith RJ, Borrell JS, Faurby S, Rajaonah MT, Randriamboavonjy T, Vorontsova MS, Cooke RSC, Phelps LN, Sayol F, Andela N, Andermann T, Andriamanohera AM, Andriambololonera S, Bachman SP, Bacon CD, Baker WJ, Belluardo F, Birkinshaw C et al (2022) Madagascar’s extraordinary biodiversity: threats and opportunities. Science 378:eadf1466. 10.1126/science.adf1466 36454830 10.1126/science.adf1466

[CR47] Rasambainarivo F, Goodman SM (2019) Disease risk to endemic animals from introduced species on Madagascar. Fowler’s Zoo Wild Anim Med Curr Ther 9:292–297. 10.1016/B978-0-323-55228-8.00043-6

[CR48] Rizor J, Yanez RA, Thaiwong T, Kiupel M (2022) *Angiostrongylus cantonensis* in a red ruffed Lemur at a zoo, louisiana, USA. Emerg Infect Dis 28:1058–1060. 10.3201/eid2805.212287 35447053 10.3201/eid2805.212287 PMC9045436

[CR49] Scheele BC, Skerratt LF, Grogan LF, Hunter DA, Clemann N, McFadden M, Newell D, Hoskin CJ, Gillespie GR, Heard GW, Brannelly L, Roberts AA, Berger L (2017) After the epidemic: ongoing declines, stabilizations and recoveries in amphibians afflicted by chytridiomycosis. Biol Conserv 206:37–46. 10.1016/j.biocon.2016.12.010

[CR50] Sears WJ, Qvarnstrom Y, Dahlstrom E, Snook K, Kaluna L, Baláž V, Fecková B, Šlapeta J, Modrý D, Jarvi S, Nutman TB (2021) AcanR3990 qPCR: a novel, highly sensitive, bioinformatically-informed assay to detect *Angiostrongylus cantonensis* infections. Clin Infect Dis 73:e1594–e1600. 10.1093/cid/ciaa1791 33252651 10.1093/cid/ciaa1791 PMC8492198

[CR51] Shanebeck KM, Lagrue C (2020) Acanthocephalan parasites in sea otters: why we need to look beyond associated mortality…. Mar Mamm Sci 36:676–689. 10.1111/mms.12659

[CR52] Šipková A, Javorská K, Anettová L, Pandian D, Cibulka P, Kačmaříková J, Novotný L, Modrý D (2025) Hedgehogs and *Angiostrongylus cantonensis*: Uncovering the role of *Atelerix albiventris* in the parasite life cycle. Integr Zool. 10.1111/1749-4877.13004 40400115 10.1111/1749-4877.13004 PMC12794780

[CR53] Spratt DM (2015) Species of *Angiostrongylus* (Nematoda: Metastrongyloidea) in wildlife: a review. Int J Parasitol Parasites Wildl 4:178–189. 10.1016/j.ijppaw.2015.02.006 25853051 10.1016/j.ijppaw.2015.02.006 PMC4381133

[CR54] Stephenson PJ, Soarimalala V, Goodman SM, Nicoll ME, Andrianjakarivelo V, Everson KM, Hoffmann M, Jenkins PD, Olson LE, Raheriarisena M, Rakotondraparany F, Rakotondravony D, Randrianjafy V, Ratsifandrihamanana N, Taylor A (2021) Review of the status and conservation of Tenrecs (Mammalia: afrotheria: Tenrecidae). ORYX 55:13–22. 10.1017/S0030605318001205

[CR55] Tesana S, Srisawangwong T, Sithithaworn P, Laha T, Andrews R (2009) Prevalence and intensity of infection with third stage larvae of *Angiostrongylus cantonensis* in mollusks from Northeast Thailand. Am J Trop Med Hyg 80:983–987 19478262

[CR56] Thompson CK, Godfrey SS, Thompson RA (2014) Trypanosomes of Australian mammals: A review. Int J Parasitol Parasites Wildl 3:57–66. 10.1016/j.ijppaw.2014.02.002 25161902 10.1016/j.ijppaw.2014.02.002 PMC4142263

[CR57] Turck HC, Fox MT, Cowie RH (2022) Paratenic hosts of *Angiostrongylus cantonensis* and their relation to human neuroangiostrongyliasis globally. One Health 15:100426. 10.1016/j.onehlt.2022.100426 36277113 10.1016/j.onehlt.2022.100426 PMC9582568

[CR58] Whittaker RJ, Fernández-Palacios JM (2007) Island biogeography: ecology, evolution, and conservation, 2nd edn. Oxford University Press, New York

[CR59] Wilkinson DA, Mélade J, Dietrich M, Ramasindrazana B, Soarimalala V, Lagadec E, le Minter G, Tortosa P, Heraud J, de Lamballerie X, Goodmanm SM, Dellagi K, Pascalis H (2014) Highly diverse morbillivirus-related *Paramyxoviruses* in wild fauna of the Southwestern Indian ocean islands: evidence of exchange between introduced and endemic small mammals. J Virol 88:8268–8277. 10.1128/jvi.01211-14 24829336 10.1128/JVI.01211-14 PMC4135957

